# Integrated analysis of transcription factor-mRNA-miRNA regulatory network related to immune characteristics in medullary thyroid carcinoma

**DOI:** 10.3389/fimmu.2022.1055412

**Published:** 2023-01-12

**Authors:** Danfeng Weng, Long He, Xiangna Chen, Huangfeng Lin, Daihan Ji, Shuting Lu, Lu Ao, Shenglin Wang

**Affiliations:** ^1^ Department of Pathology, The First Affiliated Hospital of Fujian Medical University, Fuzhou, China; ^2^ Department of Pain, The First Affiliated Hospital of Fujian Medical University, Fuzhou, China; ^3^ Department of Orthopedics, The First Affiliated Hospital of Fujian Medical University, Fuzhou, China; ^4^ Department of Bioinformatics, Fujian Key Laboratory of Medical Bioinformatics, School of Medical Technology and Engineering, Fujian Medical University, Fuzhou, China

**Keywords:** medullary thyroid carcinoma, differentially expressed genes, transcription factor, miRNA, tumor immune microenvironment

## Abstract

**Background:**

Medullary thyroid carcinoma (MTC), a thyroid C cell-derived malignancy, is poorly differentiated and more aggressive than papillary, follicular and oncocytic types of thyroid cancer. The current therapeutic options are limited, with a third of population suffering resistance. The differential gene expression pattern among thyroid cancer subtypes remains unclear. This study intended to explore the exclusive gene profile of MTC and construct a comprehensive regulatory network via integrated analysis, to uncover the potential key biomarkers.

**Methods:**

Multiple datasets of thyroid and other neuroendocrine tumors were obtained from GEO and TCGA databases. Differentially expressed genes (DEGs) specific in MTC were identified to construct a transcription factor (TF)-mRNA-miRNA network. The impact of the TF-mRNA-miRNA network on tumor immune characteristics and patient survival was further explored by single-sample GSEA (ssGSEA) and ESTIMATE algorithms, as well as univariate combined with multivariate analyses. RT-qPCR, cell viability and apoptosis assays were performed for in vitro validation.

**Results:**

We identified 81 genes upregulated and 22 downregulated in MTC but not in other types of thyroid tumor compared to the normal thyroid tissue. According to the L1000CDS2 database, potential targeting drugs were found to reverse the expressions of DEGs, with panobinostat (S1030) validated effective for tumor repression in MTC by in vitro experiments. The 103 DEGs exclusively seen in MTC were involved in signal release, muscle contraction, pathways of neurodegeneration diseases, neurotransmitter activity and related amino acid metabolism, and cAMP pathway. Based on the identified 15 hub genes, a TF-mRNA-miRNA linear network, as well as REST-cored coherent feed-forward loop networks, namely REST-KIF5C-miR-223 and REST-CDK5R2-miR-130a were constructed via online prediction and validation by public datasets and our cohort. Hub-gene, TF and miRNA scores in the TF-mRNA-miRNA network were related to immune score, immune cell infiltration and immunotherapeutic molecules in MTC as well as in neuroendocrine tumor of lung and neuroblastoma. Additionally, a high hub-gene score or a low miRNA score indicated good prognoses of neuroendocrine tumors.

**Conclusion:**

The present study uncovers underlying molecular mechanisms and potential immunotherapy-related targets for the pathogenesis and drug discovery of MTC.

## Introduction

Thyroid cancer, the most common endocrine malignancy, is mainly classified based on the histologic features into differentiated (papillary, follicular and oncocytic), medullary and anaplastic subtypes (1). Medullary thyroid carcinoma (MTC) is derived from parafollicular C cells of the thyroid gland, comprising approximately 3–5% of all thyroid cancers (2). Although less common, MTC is poorly differentiated and more aggressive than papillary, follicular and oncocytic carcinomas (3). With the combined interventions such as surgical resection and ionizing radiation, patients with MTC undergo an overall 10-year survival rate of over 70%, with 30% cases resistant to current therapies. Additionally, the 10-year survival rate for cases with distant metastasis decreases to 40% (1, 3). Therefore, comprehensive understanding of the molecular mechanisms to develop new targeted therapy is essential to better manage this malignancy.

Recent research highlights the synergistic roles of transcription factors (TFs) and miRNAs in genetic regulatory networks in forms of feed-forward or feedback loops (4). TFs are proteins that regulate gene transcription *via* binding to the promoter region of target genes (5). miRNAs, a kind of small non-coding RNAs with a length of ∼22 nucleotides, may repress the expression of target genes *via* translational inhibition or mRNA degradation (6). Tong et al. constructed a TF-miRNA-mRNA regulatory network in type 2 diabetes mellitus and breast cancer, elucidating shared mechanisms between the two diseases (7). In MTC, previous studies focused mainly on gene or miRNA profile difference between MTC and noncancerous tissue (8–10). According to the study by Minna et al., downregulated genes in MTC were partially related to thyroid function and some upregulated genes were related to neuroendocrine features (8). Romeo et al. screened plasma miR-375 as a promising prognostic marker for advanced MTC patients by microarray analysis (9). TFs are recently recognized to help accurate classification of neuroendocrine carcinomas (11). According to the study by Steiner et al., thyroid transcription factor-1 (TTF-1) was correlated with tenascin C which derived tumor proliferation in MTC. TTF-1 serves as a good marker for detecting primary or metastatic MTC (12). Another TF of yes-associated protein 1 was identified as the downstream target of miR-375 in MTC (13). Additionally, Zhang et al. constructed a regulatory network consisting of TF-miRNA and gene-miRNA associations *via* examining differentially expressed miRNAs and genes between MTC and normal thyroid tissue (10). Identification of the potential regulatory relationships between genes, TFs and miRNAs is preferred to comprehensively understand the tumorigenic mechanisms of MTC.

Since MTC arises from parafollicular C cells rather than from follicular cells in other subtypes of thyroid cancer. Uncovering the unique molecular feature of MTC favors diagnostic and therapeutic strategies of this disease. Nikiforova et al. explored differentially expressed miRNAs among thyroid cancer subtypes whereas found no overlap between MTC and other tumor subtypes (14). The differential gene expression pattern among thyroid cancer subtypes is yet to be elucidated. The present study aimed to identify the exclusive gene expression profile in MTC compared to other subtypes of thyroid neoplasms. Furthermore, we constructed a TF-mRNA-miRNA regulatory network and explored the contributions of TFs, hub genes and miRNAs to the immune characteristics of MTC and the survival of neuroendocrine tumors, so as to uncover the tumorigenic mechanisms and potential therapeutic targets of MTC.

## Materials and methods

### Public data collection

A total of 13 public datasets were used in this study. Among them, 11 datasets were downloaded from the Gene Expression Omnibus (GEO) database (http://www.ncbi.nlm.nih.gov/geo/), including 8 gene expression profiling and 3 miRNA profiling. The details of included GEO datasets were summarized in **Table 1**. Thyroid carcinoma (THCA) dataset which includes 510 tumors and 58 normal tissues was download from The Cancer Genome Atlas (TCGA) database. mRNA expression of thyroid carcinoma cells from the CCLE database was also download for validation (**Table S1**).

**Table 1:** Characteristics of the expression profile datasets from Gene Expression Omnibus database.

GEO accession	Type	Platform	Case (number)	Country	Submission date
GSE27155	Expression profiling by array	GPL96	ATC (4), MTC (2), PTC (51), FTC (13), OTC (8), FTA (10),OTA (7), normal thyroid (4)	USA	Feb 8, 2011
GSE33630	Expression profiling by array	GPL570	ATC (11), PTC (49), normal thyroid (45)	Belgium	Nov 11, 2011
GSE29265	Expression profiling by array	GPL570	ATC (9), PTC (20), normal thyroid (20)	Belgium	May 12, 2011
GSE32662	Expression profiling by array	GPL6480	MTC (52)	Spain	Oct 06, 2011
GSE29315	Expression profiling by array	GPL8300	PTC (22), FTC (9), FTA (17), OTA (9)	Belgium	May 16, 2011
GSE196264	Expression profiling by array	GPL18281	MTC (17), normal thyroid (3)	Italy	Feb 07, 2022
GSE40807	Non-coding RNA profiling by array	GPL8227	MTC (40), normal thyroid (40)	France	Sep 12, 2012
GSE97070	Non-coding RNA profiling by array	GPL18402	MTC (17), normal thyroid (3)	Italy	Mar 27, 2017
GSE30219	Expression profiling by array	GPL570	Lung tumor (293), normal lung (14)	France	Jun 26, 2011
GSE49711	Expression profiling by high throughput sequencing	GPL17553	Neuroblastoma (498)	China	Aug 09, 2013
GSE121513	Non-coding RNA profiling by RT-PCR	GPL17553	Neuroblastoma (95)	Belgium	Oct 19, 2018

### Identification of differentially expressed genes specific in MTC

To investigate MTC-specific genes, 5 gene expression datasets from the GEO database (GSE27155, GSE33630, GSE29265, GSE32662 and GSE29315) were integrated as the combined dataset with a total number of 362, which comprised of anaplastic thyroid carcinoma (ATC, n = 24), MTC (n = 54), papillary thyroid carcinoma (PTC, n = 142), follicular thyroid carcinoma (FTC, n = 22), oncocytic thyroid carcinoma (OTC, n = 8), follicular thyroid adenoma (FTA, n = 27), oncocytic thyroid adenoma (OTA, n = 16) and normal thyroid tissue (n = 69). The batch effect among the individual datasets was removed by the “sva” package in R software (version 4.1.0) (15). Firstly, the differentially expressed genes (DEGs) between the 7 tumor subtypes and normal tissue were screened out respectively *via* the “limma” package (version 3.48.3) (16) in R software. Those with adjusted P value < 0.05 and |log_2_FC| ≥ 1 were selected as DEGs. A website tool (http://bioinformatics.psb.ugent.be/webtools/Venn/) was then applied to calculate the intersections of DEGs among the tumor subtypes to identify unique biomarkers in MTC, where PTC, FTC and OTC were integrated as well differentiated thyroid carcinoma (WDTC), and FTA and OTA integrated as TA, to simplify the process. Moreover, the identified MTC-specific DEGs were further intersected with the DEGs in PTC from TCGA-THCA dataset, to obtain the final exclusive ones in MTC.

### Drug repurposing

To screen candidate therapeutic molecules for MTC, the exclusive DEGs were used as input data for an online database, the Library of Integrated Network-based Cellular Signatures (LINCS) L1000 Characteristic Direction Signature Search Engine (L1000CDS^2^) (https://maayanlab.cloud/L1000CDS2/#/index) (17). The top 50 matched compounds were returned based on the overlap of the input list with the DEGs computed from the web-based search engine.

### Functional enrichment analysis

Gene ontology (GO), including biological process, molecular function, and cellular component, and Kyoto Encyclopedia of Genes and Genomes (KEGG) pathway analyses of the DEGs were performed based on the R package “clusterProfiler” (18). Those with P value < 0.05 were considered as significantly enriched GO terms or KEGG pathways. Additionally, the R package “enrichplot” (Bioconductor) was used to generate the enrichment map and gene-concept network.

### Protein-protein interaction network and identification of hub genes

The PPI network of MTC-specific DEGs was constructed by the Search Tool for the Retrieval of Interacting Genes (STRING, version 11.5, http://string-db.org/) database (19). Those with an interaction score > 0.4 were extracted and visualized by Cytoscape (version 3.8.2) (20). Top functional modules in the network were screened by the plug-in Molecular Complex Detection (MCODE) (21). Hub genes were identified by another plug-in of cytoHubba (version 0.1) and further ranked by the maximal clique centrality (MCC) method. Default parameters were used in the above-mentioned process.

### Transcription factor-mRNA-miRNA network construction

Prior to construct the TF-mRNA-miRNA network, TFs which regulate hub genes were firstly predicted by the iRegulon plug-in in Cytoscape, where those with a normalized enrichment score (NES) > 5.0 were selected. Subsequently, miRecords (http://c1.accurascience.com/miRecords/prediction_query.php) (22) was applied to predict the regulatory miRNAs for hub genes. The Predicted Targets component of miRecords integrates 11 miRNA target prediction tools including DIANA-microT, MicroInspector, miRanda, MirTarget2, miTarget, NBmiRTar, PicTar, PITA, RNA22, RNAhybrid, and TargetScan/TargertScanS. The regulatory miRNAs predicted by at least 2 prediction tools were selected, of which those shared the downregulated miRNAs in MTC from an independent dataset (GSE40807) were considered as the targets. Differentially expressed miRNAs in GSE40807 dataset were those with adjusted P value < 0.1 and |log_2_FC| ≥ 0.58 by the “limma” package. A linear TF-mRNA-miRNA coregulation network was therefore constructed and visualized as Sankey diagram by the R package “ggalluvial”. The TF-mRNA regulatory relationship in the network was additionally investigated in multiple databases including Cistrome Data Browser (23), Genecards (version 5.12, https://www.genecards.org/), GRNdb (24) and hTFtarget (25). Furthermore, the relationships of TF-mRNA and miRNA-mRNA were validated in the datasets of GSE196264 and GSE97070 submitted by Romeo et al., which were gene and miRNA expression profiles respectively from the same MTC cohort. Additionally, TransmiR v2.0 database (26) was used to find the regulatory TFs for miRNAs in the validated linear network, where the predicted TFs common with those in the coregulation network were selected. The TF-miRNA regulation was also validated in the two datasets (GSE196264 and GSE97070). Finally, A TF-mRNA-miRNA loop for MTC was established, which was visualized by Cytoscape.

### Diagnostic verification of TFs, hub genes and miRNAs by ROC analysis

Receiver operating characteristic (ROC) analysis was performed to evaluate the diagnostic values of TFs, hub genes and miRNAs to distinguish MTC from other thyroid carcinoma subtypes or normal thyroid tissue in the combined dataset, GSE196264, GSE40807 or GSE97070, which was accomplished by R package “pROC”.

### Evaluation of tumor immune environment

To investigate the biological impact of the TF-mRNA-miRNA network, TF, hub-gene and miRNA scores for each sample were quantified based on their expression profiles using the single-sample GSEA (ssGSEA) algorithm by R package “gene set variation analysis (GSVA)” (27). Based on the mean values of TF, mRNA or miRNA scores, patients were divided into high- and low-score groups. “GSVA” package with ssGSEA method was also applied to determine the abundance of 28 tumor-infiltrating lymphocytes (TILs) in the high- and low-score groups. Additionally, the ESTIMATE score, immune score, stromal score and tumor purity in the high- and low-score groups were calculated by R package “ESTIMATE” (28). To estimate the associations of TF, hub-gene and miRNA scores with immunotherapy response, the expressions of 14 immune checkpoint genes (HAVCR2, CD48, PDCD1, CD274, PDCD1LG2, SIGLEC15, LAG3, TDO2, CTLA4, IDO1, TIGIT, LAIR1, LGALS9, TNFRSF14) (ICGs) were also analyzed in the low- and high-score groups. Moreover, the associations of individual TFs, hub genes and miRNAs with the immune characteristics were also explored.

### Patient samples

A total of 43 surgically resected thyroid specimens including non-tumor thyroid (n = 5), primary ATC (n = 4), MTC (n = 24), PTC (n = 5), and FTA (n = 5) tissues were retrospectively collected at the First Affiliated Hospital of Fujian Medical University, China. The clinicopathological parameters were presented in **Table S2**. Paraffin-embedded tissues were collected to determine the expression profiles of TFs, hub genes and miRNAs. This study was approved by the Ethics Committee of our institution. All patients signed informed consent.

### Cell culture

The human MTC cell line of TT was obtained from the Cell Bank of the Chinese Academy of Sciences (Shanghai, China). Another human MTC cell line of MZ-CRC-1 and normal thyroid cell line of Nthy-ori 3-1were provided by Dr. Chen in our institution. TT, MZ-CRC-1 and Nthy-ori 3-1 cells were cultured in F12K medium, Dulbecco’s Modified Eagle Medium (DMEM) and RPMI 1640 medium, respectively, which were supplemented with 10% fetal bovine serum (FBS) and 1% penicillin-streptomycin solution in a 37°C and 5% CO_2_ atmosphere.

### Cell viability assay

The MTC and normal thyroid cells were seeded in 96-well plates at a density of 5000 cells per well and treated with DMSO or different concentrations of panobinostat (MCE, USA) for 3 days. Cell Counting Kit-8 (CCK-8; Beyotime, Shanghai, China) solution was then added to the cells and incubated for 2 h at 37°C. The cell viability was determined at 450 nm using an ELISA plate reader instrument (ELx800, Biotek, USA).

### Apoptosis analysis

TT cells (2×10^5^/well) were incubated in 6-well plates and treated with panobinostat for 72 h. The apoptotic percentage of TT cells was measured using the Annexin V-FITC Apoptosis Detection Kit (Beyotime) and the Accuri C6 flow cytometer (BD).

### Reverse transcription quantitative polymerase chain reaction

Total RNA of formalin-fixed and paraffin-embedded (FFPE) tissues was extracted by the FFPE RNA Kit (AmoyDx, China) according to manufacturer’s protocol. TFs and hub genes were synthesized to cDNA by the FastKing-RT SuperMix (Tiangen, Beijing, China). RT-qPCR was performed by the Taq SYBR^®^ Green qPCR Premix (iScience, Lianyungang, China) and analyzed by the ABI 7500 Real-Time PCR system (Thermo Fisher Scientific Inc., MA, USA). miRNAs underwent reverse transcription and RT-qPCR by the miRcute Plus miRNA First Strand cDNA Synthesis Kit (Tiangen) and miRcute Plus miRNA qPCR Kit (Tiangen). The relative expression levels of biomarkers were calculated using 2^−△△Ct^ method and normalized by GAPDH. The primer sequences (**Table S3**) were designed and synthesized by Sangon Biotech Co. Ltd. (Shanghai, China).

### Survival analysis

Univariate survival analysis regarding the impacts of individual TFs, hub genes, miRNAs and their scores on the progression-free survival (PFS) and overall survival (OS) of patients was analyzed by the log-rank test using R package “survival” and visualized using packages “survminer” or “pheatmap”. The optimal cut-off value was determined using “survival” package to distinguish groups. Multivariate Cox regression analysis was also performed and visualized by R “forestplot” package.

### Statistical analysis

Statistical analysis was performed using R software (version 4.1.0, R Foundation for Statistical Computing, Vienna, Austria). Spearman method was used to calculate the correlation between quantitative variables by R package “psych”. Differential analysis between quantitative variables was performed using the Wilcoxon rank-sum test. P < 0.05 was considered statistically significant.

## Results

### Identification of exclusive DEGs and potential drugs in MTC

**Figure 1:**
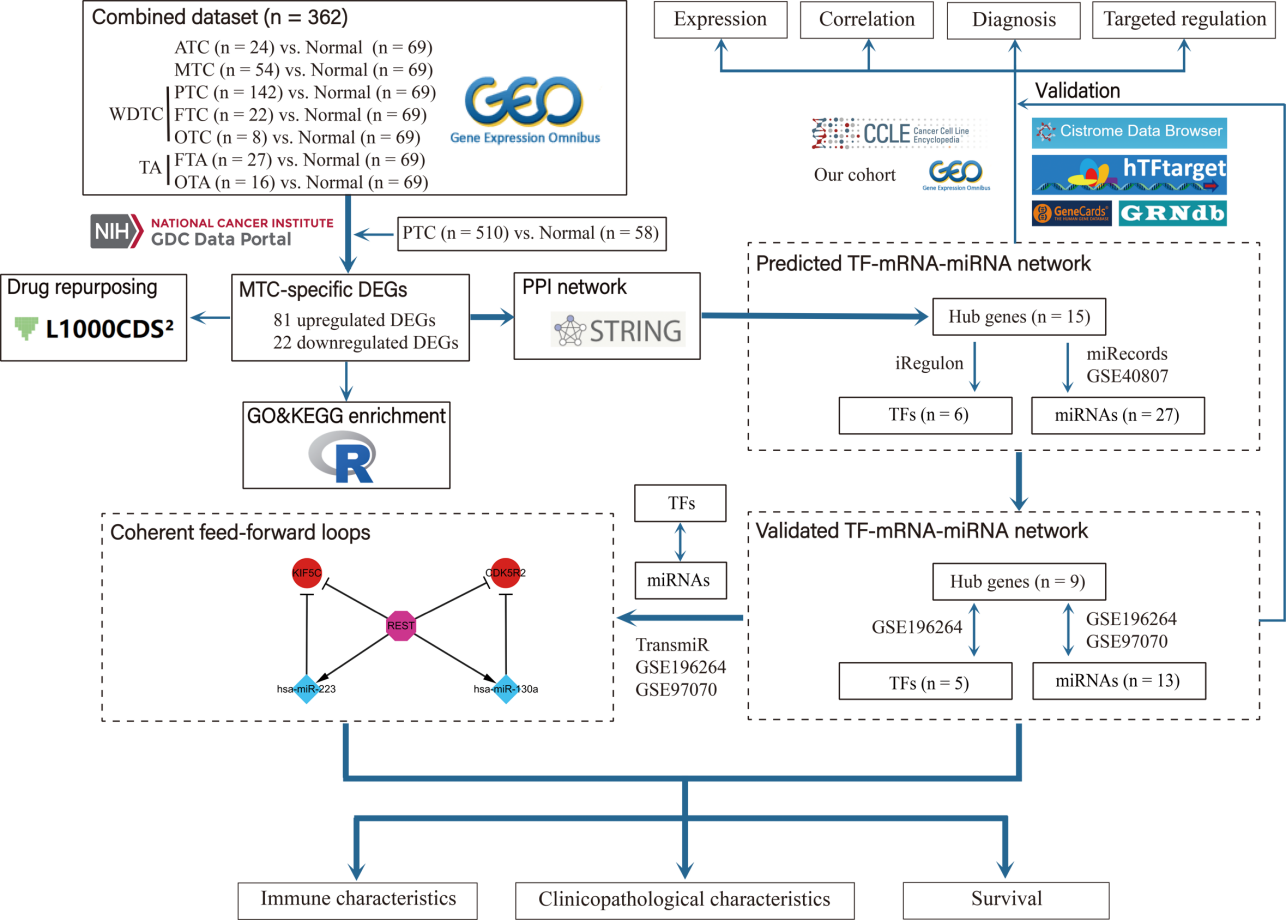
Flow chart of the study.

**Figure 2:**
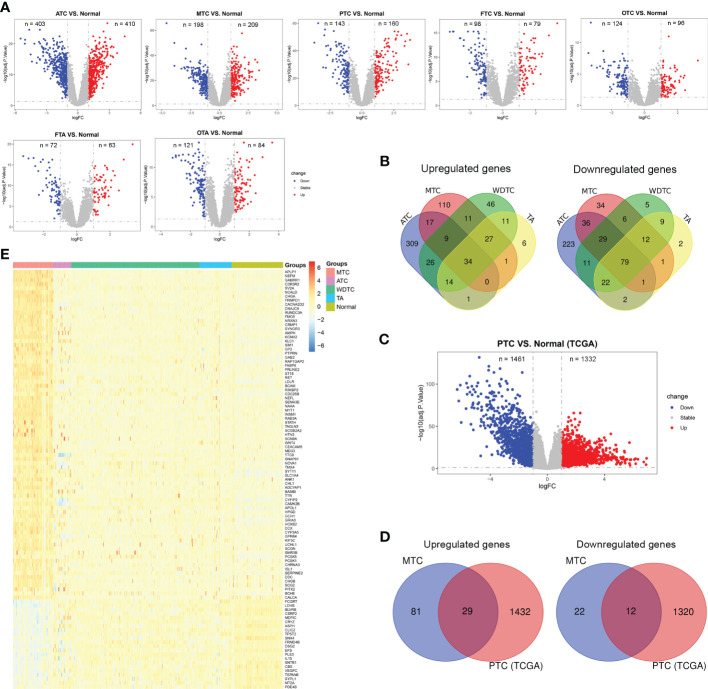
Identification of differentially expressed genes (DEGs) exclusive for MTC. **(A)** DEGs between thyroid carcinoma subtypes and normal thyroid tissue based on the combined dataset from GEO. **(B)** The intersection of DEGs among thyroid carcinoma subtypes based on the combined dataset from GEO. **(C)** DEGs between PTC and normal thyroid tissue based on the TCGA-THCA dataset. **(D)** The intersection of DEGs between MTC from GEO and PTC from TCGA. **(E)** The expression levels of 103 MTC-specific DEGs in the combined dataset. ATC, anaplastic thyroid carcinoma; MTC, medullary thyroid carcinoma; PTC, papillary thyroid carcinoma; FTC, follicular thyroid carcinoma, OTC, oncocytic thyroid carcinoma; FTA, follicular thyroid adenoma, OTA, oncocytic thyroid adenoma; WDTC, well differentiated thyroid carcinoma.

The flow chart of this study was presented in **Figure 1**. Based on the combined dataset, the DEGs in tumor subtypes compared to normal thyroid tissue were respectively investigated. ATC and MTC, the dedifferentiated and poorly differentiated subtypes of thyroid carcinoma respectively, had more DEG number than that in the well differentiated subtypes (**Figure 2A**). There were 110 upregulated and 34 downregulated genes exclusively seen in MTC (**Figure 2B**). Since PTC is the most common subtype of thyroid carcinoma, DEGs in PTC from the TCGA-THCA dataset (**Figure 2C**) was further excluded from the MTC-specific 144 DEGs. Finally, there remained 81 upregulated and 22 downregulated genes as the exclusive DEGs in MTC (**Figures 2D, E**).

Drug repurposing is an important way of approaching drug compounds, especially for the treatment of rare diseases. Based on the 103 DEGs, the L1000CDS^2^ database provided the top 50 candidates, with panobinostat (S1030) the highest score (**Figure S1A**). The CCK-8 assay confirmed that panobinostat inhibited the viability of MTC cells with lower half maximal inhibitory concentrations than normal thyroid cells (**Figure S1B**). Additionally, panobinostat increased the cell apoptotic rate of MTC (**Figure S1C**). The inhibitory effect of other repurposed drugs on MTC required further validation in the future.

### Functional enrichment of exclusive DEGs in MTC

**Figure 3:**
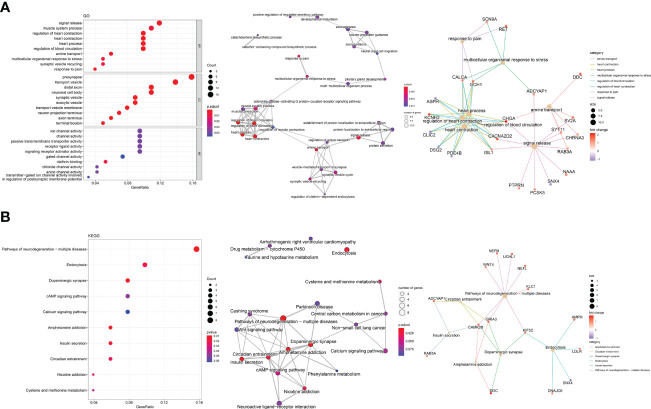
Enrichment analysis of DEGs exclusive for MTC. **(A)** The top 10 terms, enrichment map and gene-concept network by gene ontology (GO) analysis. **(B)** The top 10 pathways, enrichment map and gene-concept network by Kyoto Encyclopedia of Genes and Genomes (KEGG) pathway analysis.

The GO analysis of DEGs in MTC was presented in **Figure 3A**. The top 10 terms were mainly associated with signal release, synapse vesicle activity, amine transport, organismal response and muscle contraction (left panel), which have common genes as demonstrated in the enrichment map (middle panel). Among the shared DEGs, CALCA, GCH1, CHGA, CACNA2D2 and ISL1 were related to 5 categories or more in the gene-concept network (right panel). The top 10 KEGG pathways associated with DEGs (**Figure 3B**) were mainly pathways of neurodegeneration diseases, neurotransmitter and hormone activities and the related amino acid metabolism, cAMP pathway vital for neurodevelopment and endocrine axis, and endocytosis (left panel). All the top 10 pathways except endocytosis were closely related in the enrichment map (middle panel). According to the gene-concept network (right panel), CAMK2B, GRIA3 and KIF5C connected to the most categories among the DEGs. Additionally, NEFM, UCHL1, NEFL and KLC1 in the pathways of neurodegeneration (right panel, **Figure 3B**) were the targets of panobinostat (**Figure S1A**), indicating vital roles of this process in the development of MTC.

### PPI network and identification of hub genes in MTC

**Figure 4:**
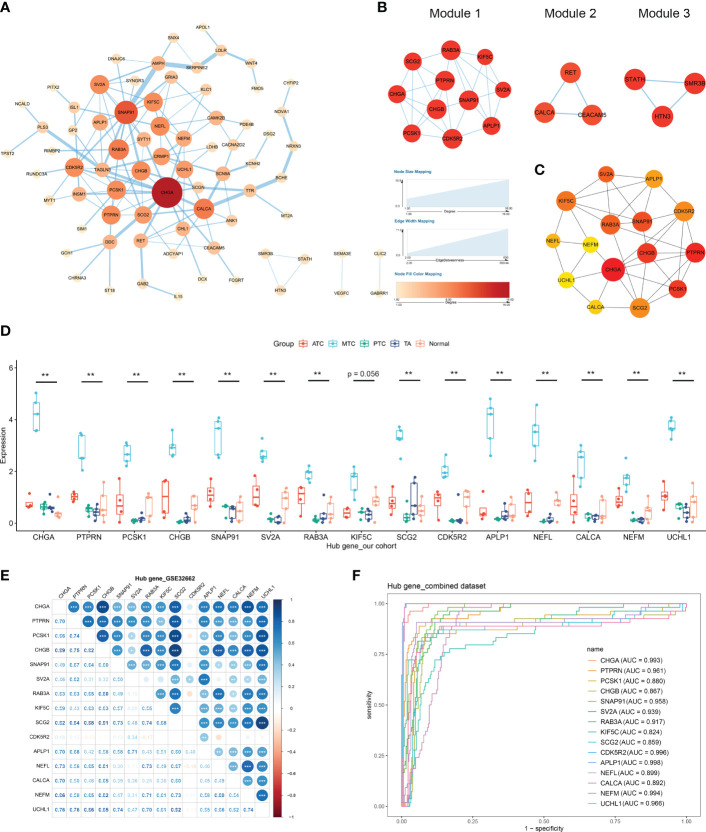
Protein–protein interaction (PPI) network construction and hub gene identification. **(A)** The PPI network of DEGs was constructed using the STRING database and visualized with Cytoscape. **(B)** Three modules identified by the MCODE algorithm. **(C)** The top 15 genes determined by the CytoHubba algorithm and ranked by the maximal clique centrality (MCC) method. **(D)** mRNA levels of hub genes by RT-qPCR in our cohort. **(E)** The correlations between hub genes by spearman analysis. **(F)** The receiver operating characteristic curve of hub genes. *p < 0.05; **p <0.01; ***p < 0.001.

The constructed PPI network consisted of 74 DEGs, including 63 upregulated and 11 downregulated ones (**Figure 4A**). Three important modules were identified from the network by MCODE, with scores of 5.8 (module 1) and 3 (module 2 and 3) (**Figure 4B**). Among the top 15 hub genes, the 11 highest ranked ones, including CHGA, PTPRN, PCSK1, CHGB, SNAP91, RAB3A, SV2A, KIF5C, CDK5R2, SCG2 and APLP1 composed module 1 (**Figure 4C**), indicating their functional connection in MTC. In our retrospective cohort, the expression levels of the hub genes were confirmed to be exclusively high in MTC but not in ATC, PTC or TA (**Figure 4D**). Additionally, based on the CCLE database, MTC cells of TT had the highest expressions of those genes compared with other thyroid carcinomas (**Figure S2A**). A consistent expression trend in MTC compared to normal thyroid tissue was also observed in another dataset of GSE196264 (**Figure S2B**). The results of spearman analysis in the GSE32662 dataset demonstrated that there were significantly positive correlations between most of the hub genes (**Figure 4E**). Similar correlation coefficient was also found in over half of the hub-gene pairs in the GSE196264 dataset although less significant (**Figure S2C**). According to the ROC analysis, every gene had an area under the curve of over 0.8 both in the combined dataset (**Figure 4F**) and GSE196264 (**Figure S2D**), indicating that those genes were promising for the differential diagnosis of medullary subtypes in THCA.

### TF-mRNA-miRNA regulatory network

**Figure 5:**
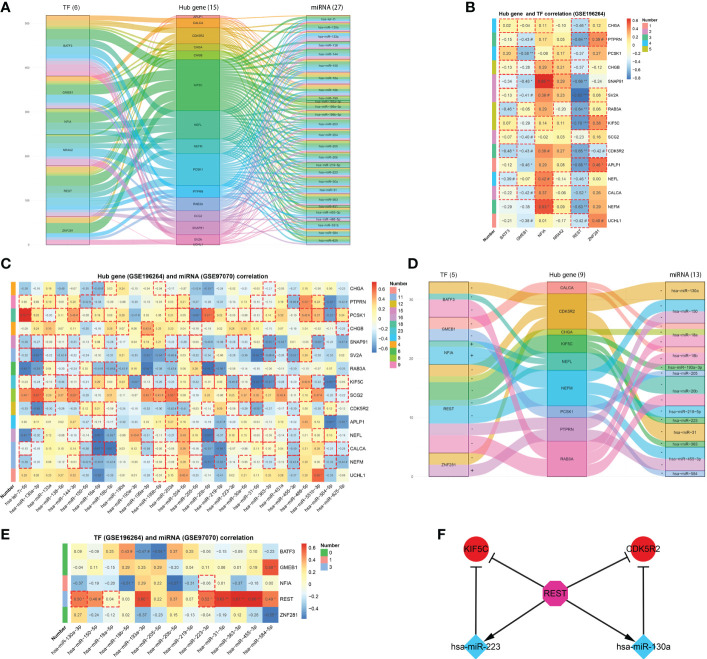
TF-mRNA-miRNA network for MTC. **(A)** Predicted TF-mRNA-miRNA network. **(B)** Heatmap of hub gene and TF correlation. **(C)** Heatmap of hub gene and miRNA correlation. **(D)** Validated TF-mRNA-miRNA network. **(E)** Heatmap of TF and miRNA correlation. **(F)** Constructed feed-forward loops based on the TF-mRNA-miRNA network. Data in the heatmap rectangle indicates correlation coefficient, of which those with dotted rectangle borders being predicted pairs. Number in the heatmap indicates pairs with dotted rectangle borders each row. #, p < 0.1; *p < 0.05; **p < 0.01; ***p < 0.001.

The iRegulon plug-in predicted 6 TFs having regulatory relationships with the hub genes, namely BATF3, GMEB1, NFIA, NR4A2, REST and ZNF281. On the other hand, the miRecords database predicted 27 miRNAs which were downregulated in MTC within the GSE40807 dataset. A predicted TF-mRNA-miRNA regulatory network was then established (**Figure 5A**), followed by validation in public databases (**Table S4**) and Romeo’s cohort. Among the TFs, REST was found to be obviously decreased in MTC (**Figures S3A, B**), which could be an excellent indicator for the diagnosis of MTC (**Figure S3D**). Based on the predicted TF-mRNA pairs, 3 of 12 pairs for BATF3, 3 of 7 pairs for GMEB1 and 9 of 12 pairs for REST were found to have a negative correlation. Whereas 2 of 5 pairs for NFIA and 3 of 6 pairs for ZNF281 have a positive correlation (**Figure 5B**). Nineteen of the 27 miRNAs were found to be significantly downregulated in MTC compared to normal tissue in the GSE97070 dataset (**Figure S3C**). All the 27 miRNAs except miR-133a and miR-584-5p presented an area under curve (AUC) of over 0.6 by ROC analysis both in the GSE40807 (**Figure S3E**) and GSE97070 datasets (**Figure S3F**). The predicted correlations of the 27 miRNAs with hub genes were then verified in Romeo’s cohort, where negatively correlated pairs were selected (**Figure 5C**). Based on the confirmed TF-mRNA and miRNA-mRNA relationships, a validated TF-mRNA-miRNA regulatory network composed of 5 TFs, 9 hub genes and 13 miRNAs was then established (**Figure 5D**) and further verified by the RT-qPCR analysis in our clinical cohort (**Figures S4A, B**). The TF-mRNA regulations in the validated network were also supported by public databases (**Table S4**). Since the aberrant expressions of TFs may mediate miRNA activity, leading to complex regulatory circuits and feedback mechanisms in cancer (29), the regulatory relationships between TFs and miRNAs in the validated network were predicted using the TransmiR v2.0 database and then validated in the GSE196264 and GSE97070 datasets (**Figure 5E**) as well as in our cohort (**Figure S4C**). As a result, two pairs of REST-miR-130a-3p and REST-miR-223-3p were found to have a significantly positive correlation. Two coherent feed-forward loops, REST-KIF5C-miR-223 and REST-CDK5R2-miR-130a were finally established, where the transcriptional repressor of REST inhibited KIF5C and CDK5R2 directly and *via* promoting the repressive effects of miRNAs on the two gene targets (**Figure 5F**).

### The correlation of TF-mRNA-miRNA network with tumor immune microenvironment

**Figure 6:**
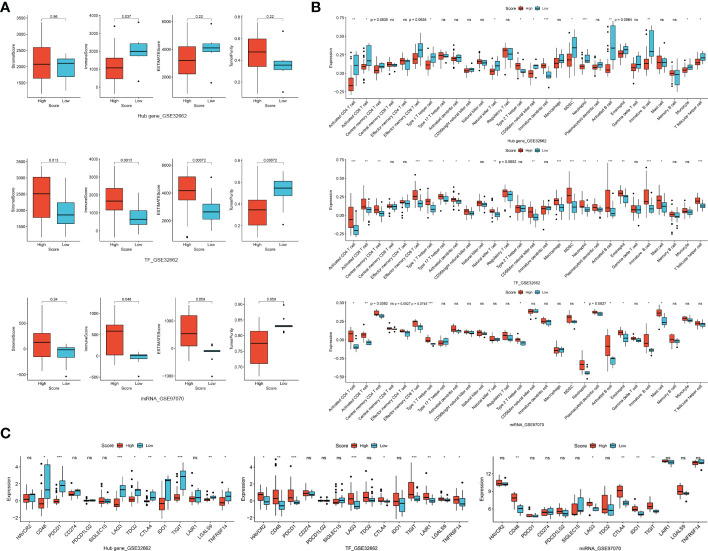
Correlation of TF, mRNA and miRNA scores in TF-mRNA-miRNA network with immune characteristics in MTC. **(A)** The relationships of hub-gene, TF and miRNA scores with stromal score, immune score, ESTIMATE score and tumor purity. **(B)** The relationships of hub-gene, TF and miRNA scores with immune infiltration levels. **(C)** The relationships of hub-gene, TF and miRNA scores with immune checkpoint genes. Ns, not significant. *p < 0.05; **p < 0.01; ***p < 0.001.

We firstly investigated the immune microenvironment in subtypes of thyroid neoplasms based on the combined dataset. MTC demonstrated low stromal, immune and ESTIMATE scores whereas relatively high tumor purity compared to other subtypes of THCA (**Figure S5A**), indicating a weak immunophenotype in the environment. Regarding immune cells and ICGs, MTC presented broadly deficient immune profiling compared to other subtypes of THCA, especially to ATC (**Figures S5B, C**). In the datasets of GSE32662 and GSE97070, patients with a low hub-gene score and with a high TF or miRNA score had a higher stromal score, immune score and ESTIMATE score whereas lower tumor purity (**Figure 6A**). In terms of immune cells, hub-gene, TF and miRNA scores were all associated with activated CD4 and CD8 T cells, central memory CD4 T cell, effector memory CD8 T cell, neutrophil, activated B cell and eosinophil (**Figure 6B**). Similarly, the association of hub-gene score with the immune cells was opposite to that of TF and miRNA scores. Regarding immunotherapy response, CD48, LAG3, CTLA4 and TIGIT were all differentially expressed between groups divided by hub-gene, TF or miRNA scores (**Figure 6C**). The associations of hub-gene and TF scores with immune microenvironment were validated in the GSE196264 dataset, where the less significance we supposed was attributed to the small sample size (**Figures S6A, B**). The results demonstrated that signatures based on the TF-mRNA-miRNA network may be excellent indicators for the immune infiltration and immunotherapy response in MTC.

**Figure 7:**
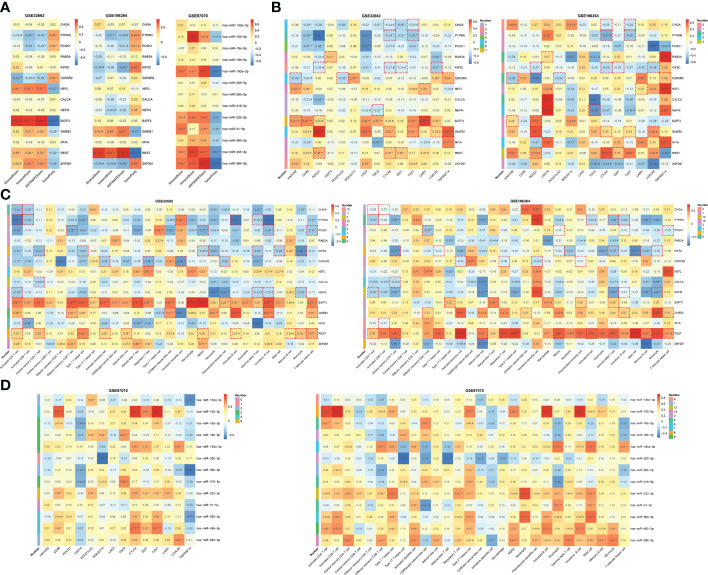
Correlation of individual biomarkers in TF-mRNA-miRNA network with immune characteristics in MTC. **(A)** The relationships of hub genes, TFs and miRNAs with stromal score, immune score, ESTIMATE score and tumor purity. **(B)** The relationships of hub genes and TFs with ICGs. **(C)** The relationships of hub genes and TFs with immune infiltration. **(D)** The relationships of miRNAs with ICGs and immune infiltration. ICGs, immune checkpoint genes. #, p < 0.1; *p < 0.05; **p < 0.01; ***p < 0.001.

At the same time, detailed correlations of individual hub genes, TFs and miRNAs with immune characteristics were analyzed (**Figure 7**). REST, the common regulator of the two feed-forward loops (**Figure 5F**), showed a positive association with stromal, immune and ESTIMATE scores whereas a negative association with tumor purity (**Figure 7A**). REST was also correlated with major ICGs of PDCD1, CTLA4 and IDO1 (**Figure 7B**). Additionally, REST was positively associated with 10 of the 28 immune cells, ranking top among all the TFs and hub genes (**Figure 7C**). KIF5C, CDK5R2 and miR-223-3p in the feed-forward loops were also tightly involved in the immune signatures (**Figures 7B-D**). Therefore, the feed-forward loop of REST-KIF5C-miR-223-3p may contribute to the immune status in MTC.

### The correlation of TF-mRNA-miRNA network with clinicopathological characteristics and survival

RET mutation, especially for M918T, is common in MTC as a driver for tumor progression, including metastasis and dismal survival (30). Patients with M918T mutation presented a relatively low hub-gene score whereas a high although not significant miRNA score (**Figure S6C**). We also observed a weak negative association between hub-gene score and lymph-node metastasis, as well as a weak positive association between miRNA score and lymph-node metastasis or lymph-node metastasis at diagnosis (**Figure S6D**). The TF score was fair between wild type and RET mutation, or between primary and lymph-node metastasis groups. Therefore, the hub-gene and miRNA signatures may contribute to the aggressive features of MTC.

**Figure 8:**
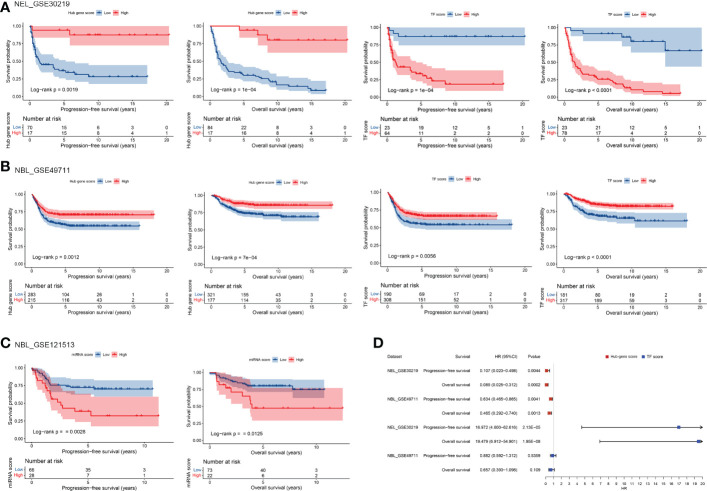
Prognostic potential of TF, mRNA and miRNA scores in TF-mRNA-miRNA network for MTC. The influence of hub-gene and TF scores on the survival of NEL **(A)** and NBL **(B)** based on univariate analysis. **(C)** The influence of miRNA score on the survival of NBL based on univariate analysis. **(D)** The influence of hub-gene and TF scores on the survival of neuroendocrine tumors with the adjustment of stromal score, immune score and tumor purity based on multivariate analysis. NEL, neuroendocrine tumor of lung; NBL, neuroblastoma.

**Figure 9:**
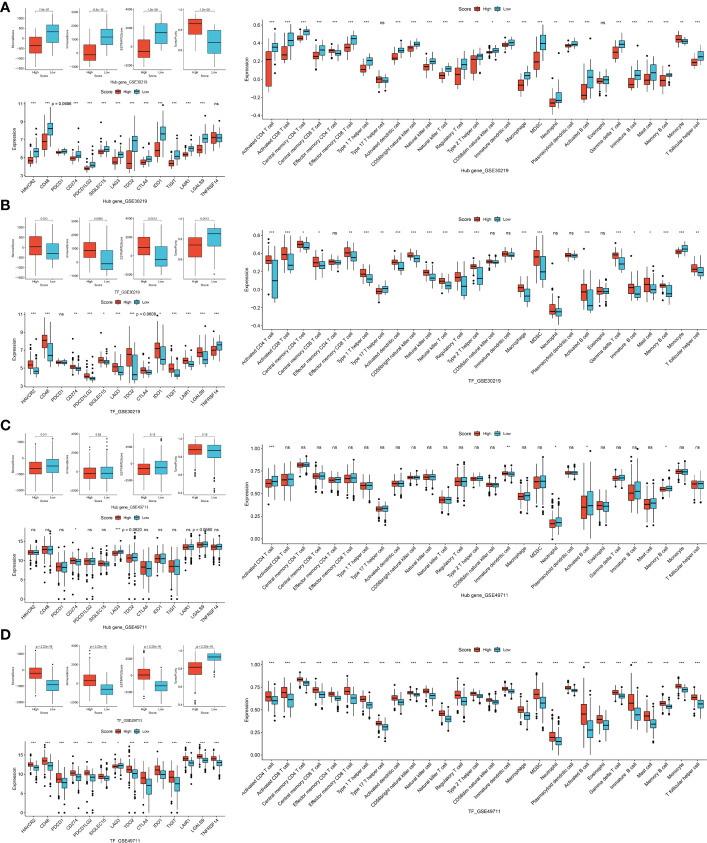
Correlation of TF, mRNA and miRNA scores in TF-mRNA-miRNA network with immune characteristics in neuroendocrine tumors. The relationships of hub-gene score **(A)** and TF score **(B)** with stromal score, immune score, ESTIMATE score, tumor purity, immune infiltration and ICGs in NEL. The relationships of hub-gene score **(C)** and TF score **(D)** with stromal score, immune score, ESTIMATE score, tumor purity, immune infiltration and ICGs in NBL. NEL, neuroendocrine tumor of lung; NBL, neuroblastoma. Ns, not significant. *p < 0.05; **p <0.01; ***p < 0.001.

**Figure 10:**
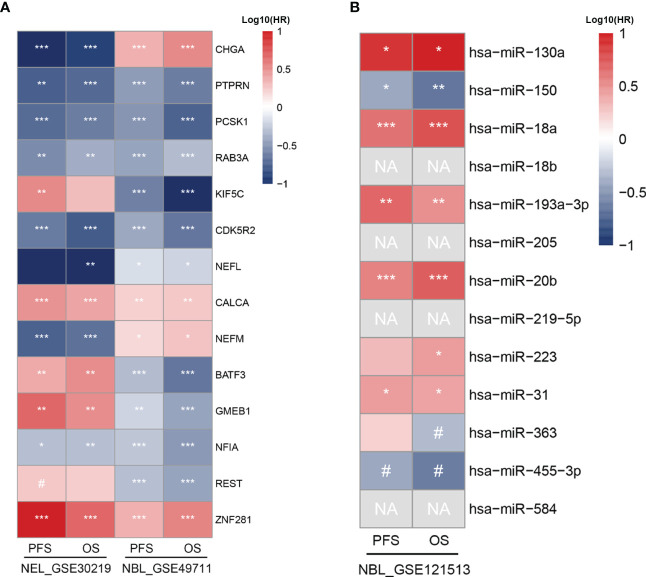
Prognostic potential of individual biomarkers in TF-mRNA-miRNA network for neuroendocrine tumors. **(A)** Heatmap of HR with significant level regarding the prognostic roles of TFs and hub genes in NEL and NBL. **(B)** Heatmap of HR with significant level regarding the prognostic roles of miRNAs in NBL. HR, hazard ratio; NEL, neuroendocrine tumor of lung; NBL, neuroblastoma; PFS, progression-free survival; OS, overall survival; NA, not available. ^#^p < 0.1; *p < 0.05; **p < 0.01; ***p < 0.001.

To evaluate the prognostic value of TF-mRNA-miRNA network, 3 cohorts including neuroendocrine tumor of lung (NEL, GSE30219) and neuroblastoma (NBL, GSE49711 and GSE121513), which share the neuroendocrine properties with MTC, were included in this study. Univariate analysis demonstrated that the hub-gene score was positively associated with the PFS and OS time both in NEL and NBL (**Figures 8A, B**). The TF score had a negative association with the PFS and OS time in NEL whereas a positive association with the survival in NBL (**Figures 8A, B**). Since miR-18b, miR-205, miR-219-5p and miR-584 were not available in the GSE121513 dataset, miRNA score in NBL was calculated based on the rest 9 counterparts. The miRNA score was found to negatively correlate with the PFS and OS time in NBL (**Figure 8C**). Additionally, there was a negative association of the hub-gene score (**Figures 9A, C**), as well as a positive association of the TF score (**Figures 9B, D**) with immune characteristics both in NEL and NBL, similar with those in MTC. Considering the contribution of tumor environment to prognosis in cancer, stromal score, immune score and tumor purity were included to explore the independent role of hub-gene and TF scores for the outcome of neuroendocrine tumors. As demonstrated in **Figure 8D**, the hub-gene score was a protective factor for the PFS and OS time both in NEL and NBL independent of tumor environment characteristics. Regarding TF score, the independent contribution to worse prognosis was observed in NEL but not in NBL. The prognostic values of individuals in the TF-mRNA-miRNA network were also investigated and presented in **Figure 10**. Specifically, CDK5R2, KIF5C and REST were protective factors for the survival of NBL (**Figure 10A**). Whereas miR-130a and miR-223-3p were adverse factors for the outcome of NBL (**Figure 10B**).

## Discussion

Medullary thyroid cancer (MTC) is transformed from neural crest–derived thyroid C cells, with distinct features from other types of thyroid cancer (31). Investigation of the unique molecular characteristics favors improved understanding of the tumorigenesis of MTC. We here explored exclusive aberrant gene expression profile in MTC and found potential targeting drugs reversing their expressions. Fifteen hub genes were identified and verified to well differentiate MTC from other subtypes of thyroid neoplasms. Moreover, a TF-mRNA-miRNA linear network, as well as REST-cored coherent feed-forward loop networks, namely REST-KIF5C-miR-223 and REST-CDK5R2-miR-130a were constructed and validated in MTC. Hub-gene, TF and miRNA scores by the ssGSEA method in the TF-mRNA-miRNA network were involved in the tumor immune characteristics of the neuroendocrine tumors of MTC, NBL and NEL. Additionally, a high hub-gene score and a low miRNA score indicated good prognoses of neuroendocrine tumors.

In this study, 103 DEGs specific for MTC were identified from multiple public datasets (**Figure 2E**), which were involved in synapse cell activity, neurotransmitter release, neurodegeneration diseases, calcium signaling pathway, wnt and cAMP pathways. A previous study reported the hyperactivation of proteins related to neurodegeneration in MTC, whereas their inhibition decreased the tumor growth (32). According to our study, panobinostat could suppress the cell viability and induce apoptosis in MTC *via* the pathways of neurodegeneration. As an inhibitor of histone deacetylases, the antitumor effect of panobinostat on other neuroendocrine tumors was also reported (33, 34). The disruption of wnt pathway exerts antitumor activity against MTC *via* the reduction of RET expression (35). cAMP signaling triggers the synthesis and secretion of calcitonin, a well-known marker secreted by the parafollicular cells in MTC (36). Of the 15 hub genes, CHGA, CHGB, SNAP91, SCG2, SV2A, APLP1, UCHL1, PTPRN, PCSK1, RAB3A and CALCA were largely detected in cancers with the neuroendocrine feature (37–41). KIF5C, CDK5R2, NEFL and NEFM mainly contribute to neuronal activity. The encoded protein levels of 10 hub genes in MTC were also observed to be elevated compared to normal tissue by Zhan et al. (42). The exclusive expressions of the other five hub genes, namely SNAP91, APLP1, PTPRN, PCSK1 and CDK5R2 in MTC were firstly reported in this study. Our present study, together with the study by Zhan et al., indicated the involvement of synaptic vesicle activity in the pathological process of MTC (42).

It is controversial regarding immune infiltration in MTC. According to Bongiovanni et al., there was immunohistochemically low PD-L1 expression in both the tumor cells and immune cells in MTC (43). Whereas a recent study observed more expressions of ICGs in a large cohort of MTC cases, which were associated with tumor recurrence (44). Pozdeyev et al. also found broad expressions of immune cells and immune regulatory molecules in MTC (45). The present study identified broadly deficient immune profiling in MTC compared to other subtypes of THCA (**Figure S5A-C**). Additionally, the study found that tumors with a high hub-gene score, low TF and miRNA scores tended to have high tumor purity, low immune and stromal scores, low infiltration levels of activated CD4 and CD8 T cells, central memory CD4 T cell, effector memory CD8 T cell, neutrophil, activated B cell and eosinophil, as well as decreased expressions of exhausted immune markers including CD48, LAG3, CTLA4 and TIGIT. Among the hub genes, PTPRN expression was reported to significantly restraint the infiltrating levels of CD4 T cells, B cells, macrophages, neutrophil, and dendritic cells in low grade glioma (46). The negative regulation between PTPRN expression and immune response as well as immune cells was also observed in lung adenocarcinoma (47). In glioma, PCSK1 knockdown could switch macrophages toward an antitumor immunophenotype and induce tumor regression (48). Apart from hub genes, TFs were also reported to be involved in immune microenvironment. BATF3 overexpression promotes the memory response of CD8 T cells, whereas its deficiency impacts on CD8-T-cell survival and fitness (49). Moreover, BATF3 as a T cell receptor is required for dendritic cells to recruit effector CD8 T cells within the tumor environment, and the absence of dendritic cells in β-catenin-expressing tumors results in failed T cell migration into tumors (50). GMEB1 is capable of binding to apoptosis-related caspases to protect against glucocorticoid-induced T cell apoptosis (51). REST, the core regulator of the feed-forward loops in this study, was also found to correlate with activated CD4 T cells in neurodegeneration disease (52). Among the 13 miRNAs in the network, miR150 and miR‐193a are upregulated during the activation of T cells (53, 54). According to Kumar et al, miR-455-3p is upregulated in the B lymphocytes and could be a potential biomarker for Alzheimer’s disease (55). As a member of the feed-forward loop of REST-KIF5C-miR-223, miR-223 is principally expressed in myeloid cells, controlling the activation of neutrophil. miR-223 gene knockdown may induce increased susceptibility to infection as a consequence of insufficient neutrophil recruitment (56). Consistently, this study observed a close relationship between miR-223 and neutrophil (**Figure 7D**). Our study for the first time found the associations of neuroendocrine- and neuron-related genes as well as their regulatory TFs and miRNAs with immune characteristics in MTC and in other neuroendocrine tumors of NEL and NBL. According to Schoot et al., MTC patients receiving dendritic cell vaccination therapy revealed a calcitonin- and CEA-specific immunoreactivity of T-cell response, leading to tumor regression (57). A clinical trial reported promising benefit from the combined treatment of dendritic cell vaccination and anti-CEA vaccine in MTC (58). Therefore, treatment targeting the biomarkers identified in this study may help improve the immunotherapeutic response in MTC as well as other neuroendocrine malignancies.

In order to explore the prognostic value of TF-mRNA-miRNA network in MTC, we extended our analysis to other neuroendocrine tumors of NEL and NBL. A high hub-gene score and a low miRNA score indicated favorable prognoses both in NEL and NBL. The TF score demonstrated a negative impact on the survival of NEL but a positive impact on the survival of NBL. According to a previous study by Liu et al., activated neuroendocrine markers including CHGA predicted good progression-free and overall survival for patients with well-differentiated pancreatic neuroendocrine neoplasms (59). Another neuroendocrine marker of RAB3A also correlates with better overall survival in pancreatic cancer (60). In hepatocellular carcinoma, patients with a low expression of CDK5R2 in tumor displayed poor overall survival (61). PTPRN expression, which negatively regulates immune cell infiltration in low grade glioma, is correlated with a favorable prognosis of patients (46). Another study also reported high immune, stromal and ESTIMATE scores whereas low tumor purity in low-grade-glioma patients with dismal survival (62). Consistently, our results found a negative association of the hub-gene signature with immune characteristics but positive associations with tumor purity and clinical outcome of neuroendocrine tumors. Moreover, with the adjustment of stromal score, immune score and tumor purity, the hub-gene signature also played a protective role for the survival of NEL and NBL. More mechanisms of the hub genes on the prognoses of neuroendocrine tumors require further exploration in the future. The opposite impact of TF score on the survival of NEL and NBL may attributed to BATF3, GMEB1 and REST, since NFIA and ZNF281 showed uniform influence on the survival of the two neuroendocrine tumors. In astrocytoma, the high expression of NFIA contributes to improved progression-free survival and overall survival for patients (63). On the contrary, ZNF281 expression is associated with a worse prognosis in human soft tissue sarcomas (64). Two miRNAs in the feed-forward loops, miR-130a and miR-223, both predicted short survival time for patients with NBL in this study. In primary gastrointestinal diffuse large B-cell lymphoma, patients with an increased expression of miR-130a tend to suffer worse survival compared to those with a low miR-130a level (65). Additionally, a high level of circulating miR-130a is also correlated with a poor prognosis in patients with colorectal cancer (66). According to a previous study, overexpressed miR-223-3p promoted the migration and invasion of breast cancer cells and was associated with a poor prognosis in patients (67). The association of a high miR-455-5p level with favorable survival in patients was previously observed in MTC in the study by Zheng et al. (68). Consistently, our study observed a protective prognostic value of miR-455 for NBL, confirming its influence for the outcome of neuroendocrine tumors.

To conclude, the present study identified MTC-specific DEGs by integrated analysis and found 15 hub genes. Additionally, we established a TF-mRNA-miRNA linear network composed of 5 TFs, 9 hub genes and 13 miRNAs, as well as two coherent feed-forward loops of REST-KIF5C-miR-223 and REST-CDK5R2-miR-130a. The hub-gene, TF and miRNA signatures were closely related to tumor immune characteristics of MTC. Moreover, a high hub-gene score or a low miRNA score indicated favorable prognoses of neuroendocrine tumors. TFs, hub genes and miRNAs in the TF-mRNA-miRNA network may serve as tumorigenic biomarkers and therapeutic targets for MTC. Further experiments are required to validate our findings and uncover their potential molecular mechanisms for MTC.

## Data availability statement

The original contributions presented in the study are included in the article/**Supplementary Material**. Further inquiries can be directed to the corresponding authors.

## Ethics statement

The studies involving human participants were reviewed and approved by the Ethics Committee of First Affiliated Hospital of Fujian Medical University. The patients/participants provided their written informed consent to participate in this study.

## Author contributions

DW and LH drafted the manuscript. XC and HL conducted the *in vitro* experiments. DJ and SL acquired data and performed the analysis and data interpretation. LA and SW designed the work and revised the manuscript. All authors contributed to the article and approved the submitted version.

## Supplementary material

Supplementary Figure 1 Predicted drugs for MTC and *in vitro* validation. **(A)** Predicted reagents and their reversed DEG signature in MTC by the L1000CDS^2^ database. The red label-bars in columns indicate the score of compounds in descending order. The red and blue label-bars in left-sided rows indicate the upregulated and downregulated input signature. The blue and red squares in the matrix indicate the reversed effect of respective compounds on the input signature. **(B)** S1030 (panobinostat) effect on the viability of MTC and normal thyroid cells by Cell Counting Kit-8 assay. **(C)** S1030 (panobinostat) effect on the apoptosis of TT cells by flow cytometry analysis. ***, p < 0.001.

Supplementary Figure 2 Expression and diagnostic values of hub genes. Expression levels of hub genes in THCA subtypes and normal thyroid tissue in the CCLE database **(A)** and GSE196264 **(B)** dataset. **(C)** The expressional correlations between hub genes in MTC based on spearman analysis. **(D)** The receiver operating characteristic curve analysis for the differential diagnosis of hub genes. *, p < 0.05; **, p < 0.01; ***, p < 0.001.

Supplementary Figure 3 Expression and diagnostic values of TFs and miRNAs in the predicted network. Expression levels of TFs in THCA subtypes and normal thyroid tissue in the GSE196264 dataset **(A)** and CCLE database **(B)**. **(C)** Expression levels of miRNAs in MTC and normal thyroid tissue based on the GSE97070 dataset. The receiver operating characteristic curve analysis for the differential diagnoses of TFs based on the GSE196264 dataset **(D)**, as well as of miRNAs based on the GSE40807 **(E)** and GSE97070 datasets **(F)**. Ns, not significant. *, p < 0.05; **, p < 0.01.

Supplementary Figure 4 Validated correlations between biomarkers in TF-mRNA-miRNA network in MTC by RT-qPCR. **(A)** Heatmap of hub gene and TF correlation in our cohort. **(B)** Heatmap of hub gene and miRNA correlation in our cohort. **(C)** Heatmap of TF and miRNA correlation in our cohort. Data in the heatmap rectangle indicates correlation coefficient, of which those with dotted rectangle borders being targeted pairs. Number in the heatmap indicates pairs with dotted rectangle borders each row. #, p < 0.1; *, p < 0.05; **, p < 0.01; ***, p < 0.001.

Supplementary Figure 5 Immune environment in thyroid tissues. **(A)** The stromal score, immune score, ESTIMATE score and tumor purity in THCA subtypes and normal tissue in the combined dataset. **(B)** The immune infiltration levels in THCA subtypes and normal tissue in the combined dataset. **(C)** The levels of immune checkpoint genes in THCA subtypes and normal tissue in the combined dataset.

Supplementary Figure 6 Correlations of TF, mRNA and miRNA scores in TF-mRNA-miRNA network with immune characteristics and clinical characteristics in MTC. **(A)** The relationship of hub-gene score with stromal score, immune score, ESTIMATE score, tumor purity, immune infiltration and ICGs. **(B)** The relationship of TF score with stromal score, immune score, ESTIMATE score, tumor purity, immune infiltration and ICGs. The relationships of hub-gene, TF and miRNA scores with RET mutation **(C)** as well as lymph-node metastasis **(D)**.
