# Supplementary figures and images for: Integrated analysis of transcription factor-mRNA-miRNA regulatory network related to immune characteristics in medullary thyroid carcinoma
Source: Front Immunol. 2023 Jan 12;13:1055412. doi: 10.3389/fimmu.2022.1055412 (PMC9877459; doi:10.3389/fimmu.2022.1055412)

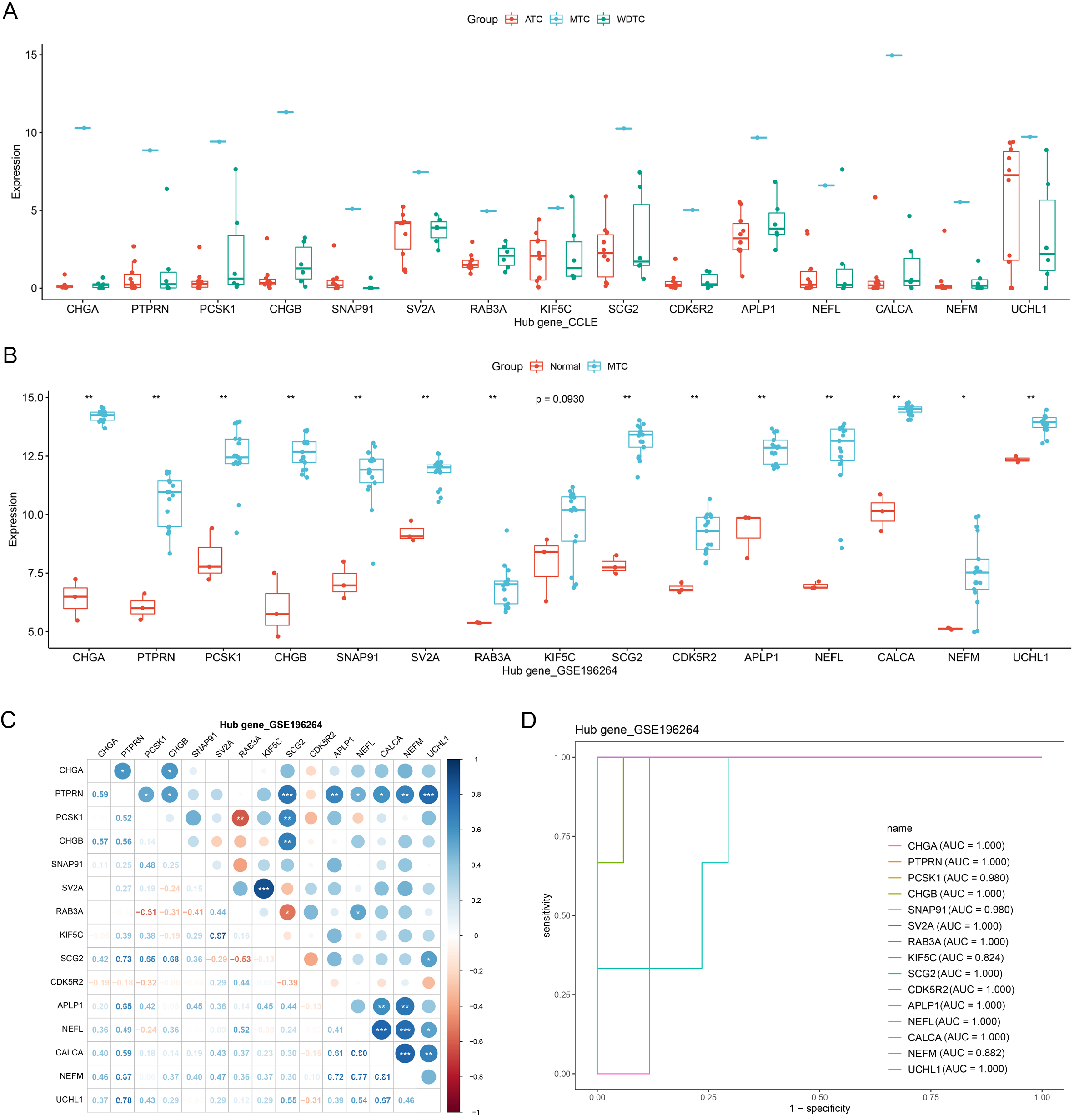

Supplement: Supplementary Figure 2 — Expression and diagnostic values of hub genes. Expression levels of hub genes in THCA subtypes and normal thyroid tissue in the CCLE database (A) and GSE196264 (B) dataset. (C) The expressional correlations between hub genes in MTC based on spearman analysis. (D) The receiver operating characteristic curve analysis for the differential diagnosis of hub genes. *, p < 0.05; **, p < 0.01; ***, p < 0.001. [file Image_2.tif]

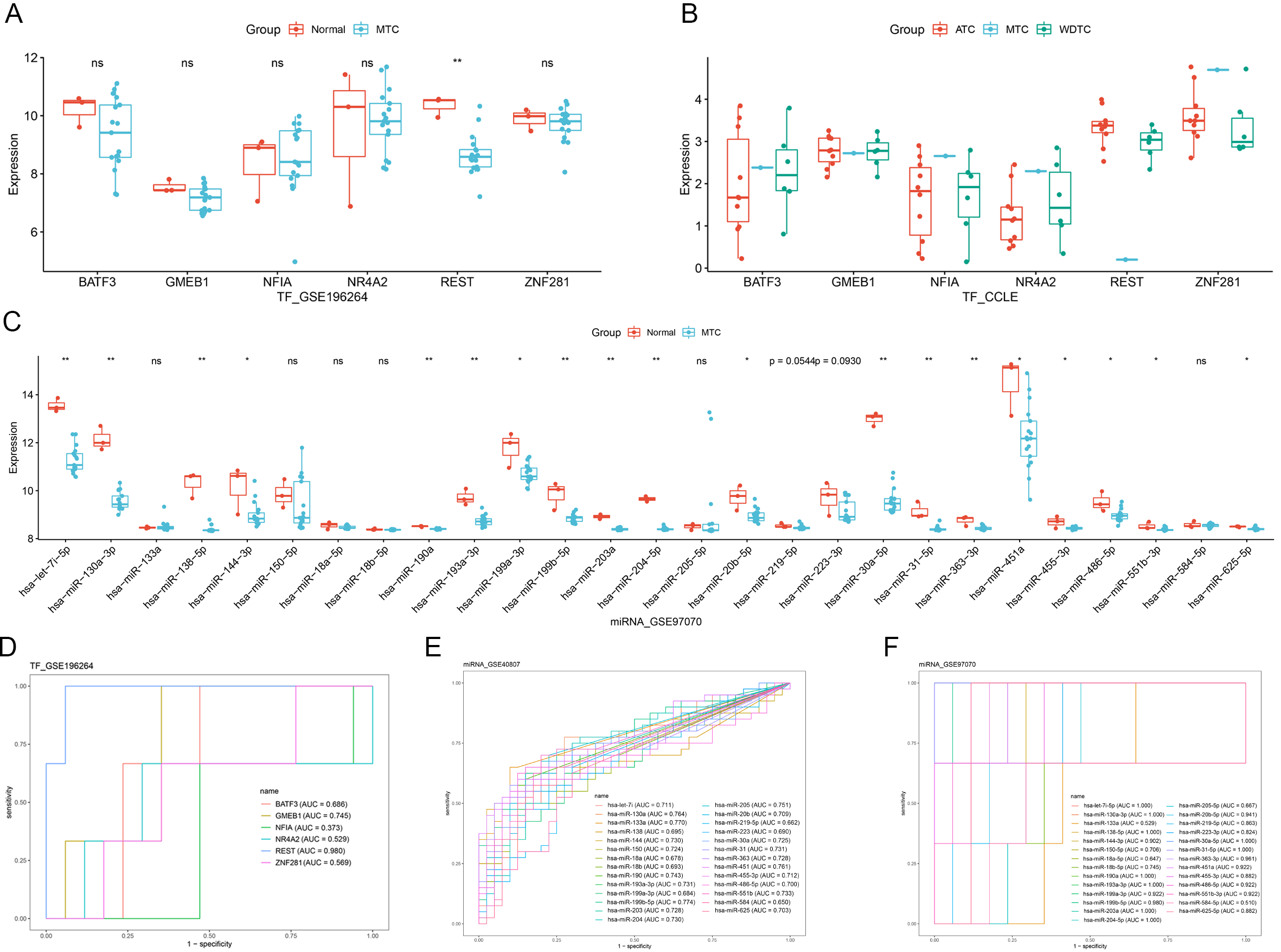

Supplement: Supplementary Figure 3 — Expression and diagnostic values of TFs and miRNAs in the predicted network. Expression levels of TFs in THCA subtypes and normal thyroid tissue in the GSE196264 dataset (A) and CCLE database (B). (C) Expression levels of miRNAs in MTC and normal thyroid tissue based on the GSE97070 dataset. The receiver operating characteristic curve analysis for the differential diagnoses of TFs based on the GSE196264 dataset (D), as well as of miRNAs based on the GSE40807 (E) and GSE97070 datasets (F). Ns, not significant. *, p < 0.05; **, p < 0.01. [file Image_3.tif]

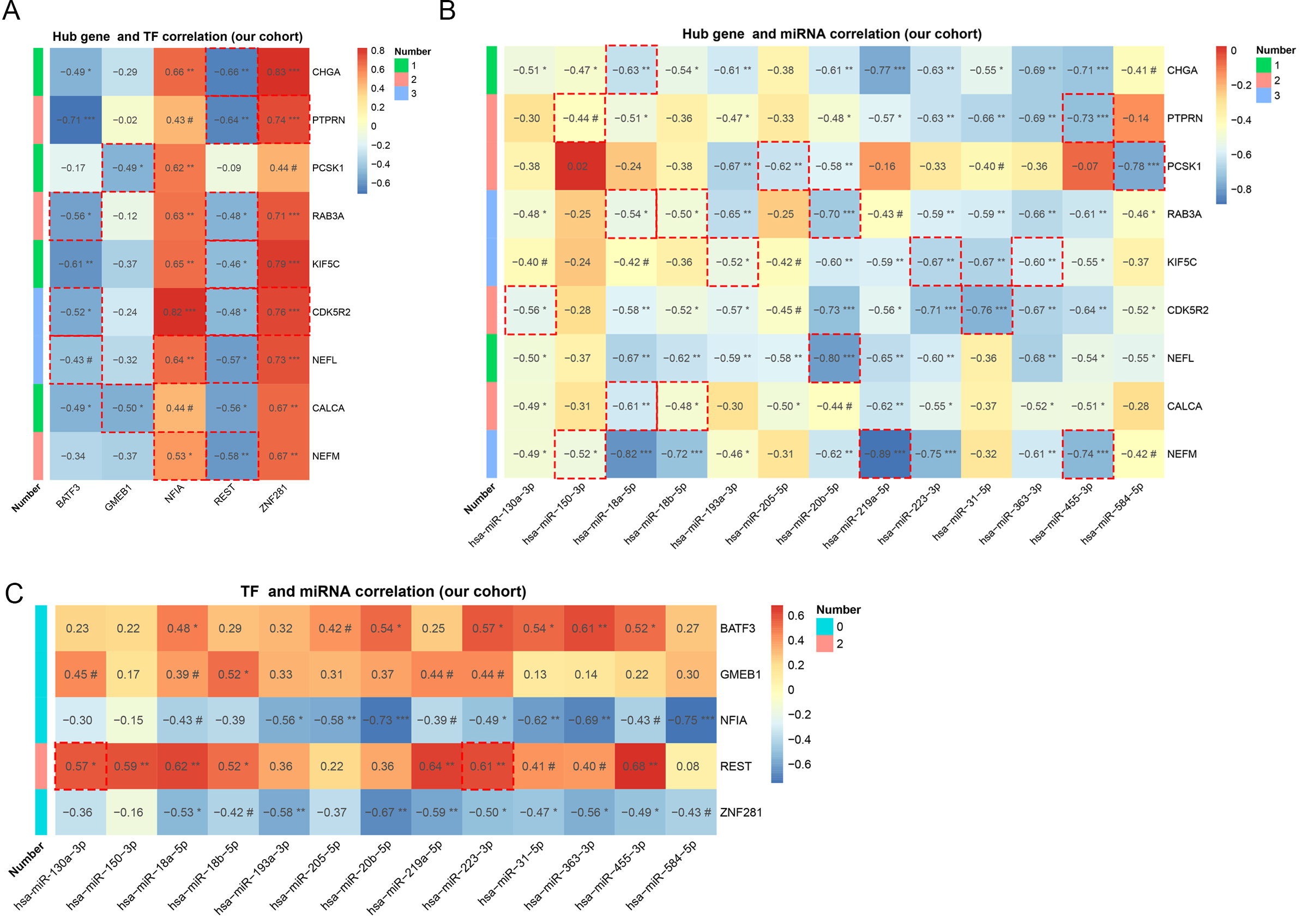

Supplement: Supplementary Figure 4 — Validated correlations between biomarkers in TF-mRNA-miRNA network in MTC by RT-qPCR. (A) Heatmap of hub gene and TF correlation in our cohort. (B) Heatmap of hub gene and miRNA correlation in our cohort. (C) Heatmap of TF and miRNA correlation in our cohort. Data in the heatmap rectangle indicates correlation coefficient, of which those with dotted rectangle borders being targeted pairs. Number in the heatmap indicates pairs with dotted rectangle borders each row. #, p < 0.1; *, p < 0.05; **, p < 0.01; ***, p < 0.001. [file Image_4.tif]

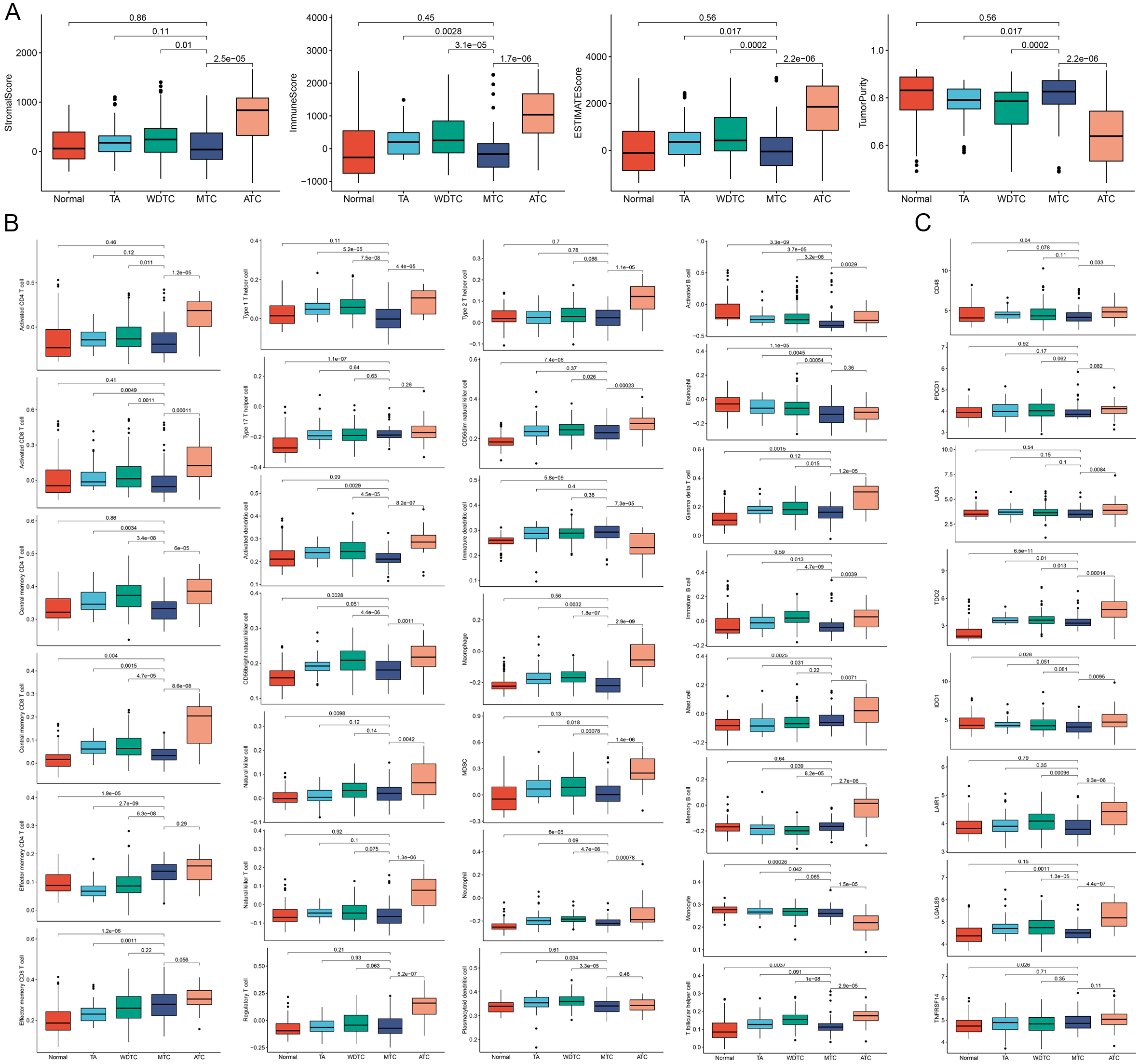

Supplement: Supplementary Figure 5 — Immune environment in thyroid tissues. (A) The stromal score, immune score, ESTIMATE score and tumor purity in THCA subtypes and normal tissue in the combined dataset. (B) The immune infiltration levels in THCA subtypes and normal tissue in the combined dataset. (C) The levels of immune checkpoint genes in THCA subtypes and normal tissue in the combined dataset. [file Image_5.tif]

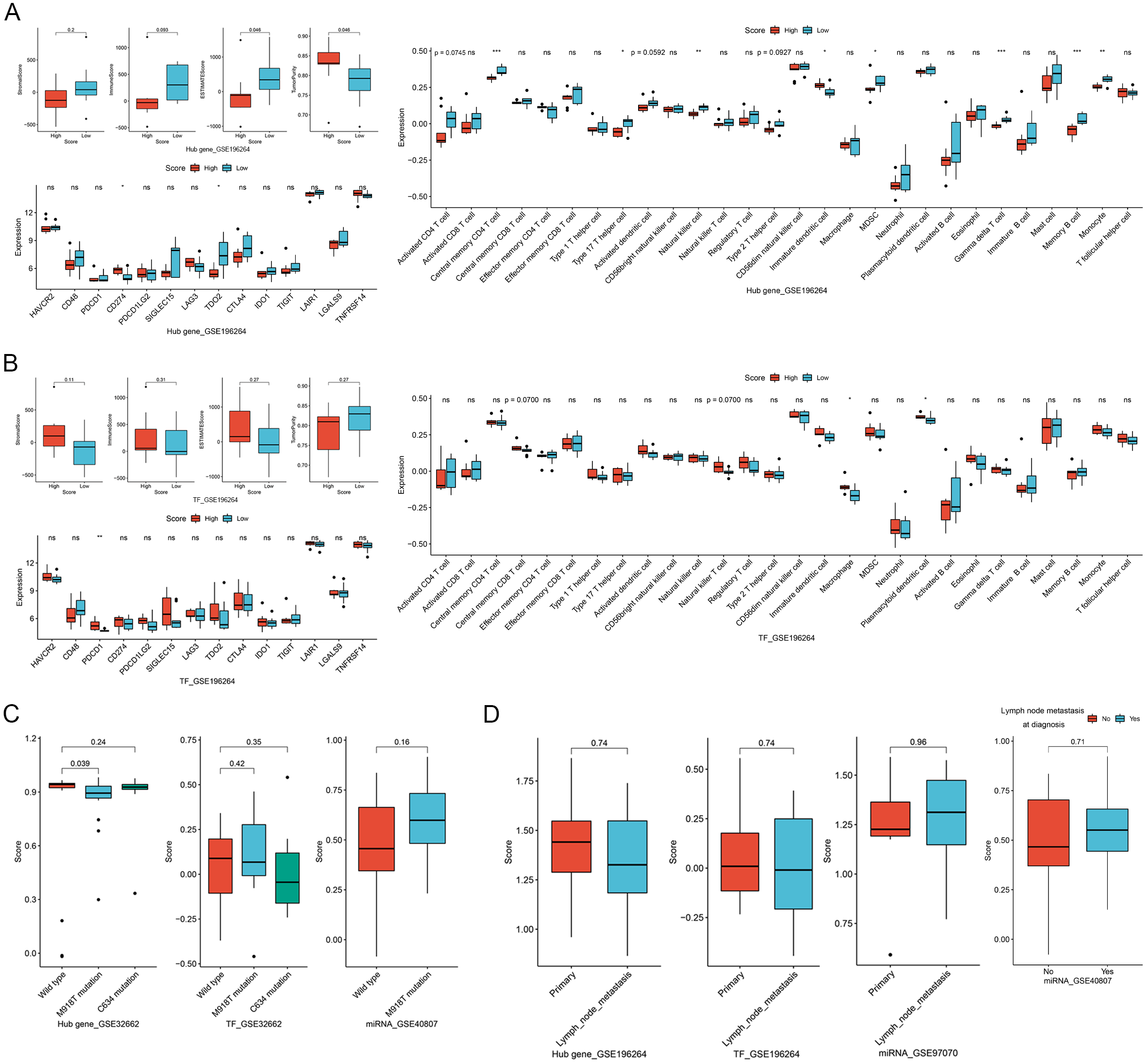

Supplement: Supplementary Figure 6 — Correlations of TF, mRNA and miRNA scores in TF-mRNA-miRNA network with immune characteristics and clinical characteristics in MTC. (A) The relationship of hub-gene score with stromal score, immune score, ESTIMATE score, tumor purity, immune infiltration and ICGs. (B) The relationship of TF score with stromal score, immune score, ESTIMATE score, tumor purity, immune infiltration and ICGs. The relationships of hub-gene, TF and miRNA scores with RET mutation (C) as well as lymph-node metastasis (D). [file Image_6.tif]
