# Supplementary material for: Integrated analysis of transcription factor-mRNA-miRNA regulatory network related to immune characteristics in medullary thyroid carcinoma
Source: Front Immunol. 2023 Jan 12;13:1055412. doi: 10.3389/fimmu.2022.1055412 (PMC9877459; doi:10.3389/fimmu.2022.1055412)
Supplement: Supplementary file 8 [file Table_1.doc]

| **Table S1** Characteristics of the dataset from the CCLE database | | | |
| --- | --- | --- | --- |
| ID | Cells | Subtype | Dataset |
| ACH-001306 | 8305C | ATC | Expression 22Q2 Public subset |
| ACH-001307 | 8505C | ATC |
| ACH-001443 | ASH3 | ATC |
| ACH-000191 | BHT101 | ATC |
| ACH-000174 | CAL62 | ATC |
| ACH-000903 | FTC133 | ATC |
| ACH-002041 | HOTHC | ATC |
| ACH-001356 | MB1 | ATC |
| ACH-000163 | SW579 | ATC |
| ACH-002042 | T3M5 | ATC |
| ACH-001321 | TT | MTC |
| ACH-000456 | BCPAP | PTC |
| ACH-001528 | IHH4 | PTC |
| ACH-000897 | FTC238 | FTC |
| ACH-000058 | ML1 | FTC |
| ACH-001384 | RO82W1 | FTC |
| ACH-000716 | TT2609C02 | FTC |
