# Supplementary material for: Integrated analysis of transcription factor-mRNA-miRNA regulatory network related to immune characteristics in medullary thyroid carcinoma
Source: Front Immunol. 2023 Jan 12;13:1055412. doi: 10.3389/fimmu.2022.1055412 (PMC9877459; doi:10.3389/fimmu.2022.1055412)
Supplement: Supplementary file 9 [file Table_2.doc]

| **Table S2** Clinicopathological characteristics of our cohort | | | | | |
| --- | --- | --- | --- | --- | --- |
| Variable | ATC (n = 4) | MTC (n = 24) | PTC (n = 5) | FTA (n = 5) | Normal (n = 5) |
| Sex |  |  |  |  |  |
| Male | 2 (50%) | 13 (54.2%) | 2 (40%) | 3 (60%) | 3 (60%) |
| Female | 2 (50%) | 11 (45.8%) | 3 (60%) | 2 (40%) | 2 (40%) |
| Age (years), median (range) | 66.5 (54-72) | 57 (31-67) | 55 (42-64) | 52 (40-65) | 47 (36-62) |
| Tumor size (cm) | 4.80 ± 0.88 | 1.68 ± 1.02 | 1.24 ± 0.28 | 0.74 ± 0.14 | - |
| Bilateral distribution |  |  |  |  |  |
| Yes | 2 (50%) | 3 (12.5%) | 0 | 0 | - |
| No | 2 (50%) | 21 (87.5%) | 5 (100%) | 5 (100%) | - |
| TNM stage |  |  |  |  |  |
| I/II | 0 | 6 (25%) | 2 (40%) | - | - |
| III | 0 | 6 (25%) | 2 (40%) | - | - |
| IV | 4 (100%) | 12 (50%) | 1 (20%) | - | - |
| Invasion (vascular/capsular) |  |  |  |  |  |
| Yes | 4 (100%) | 9 (37.5%) | 1 (20%) | 0 | - |
| No | 0 | 15 (62.5%) | 4 (80%) | 5 (100%) | - |
| Metastasis |  |  |  |  |  |
| Yes | 2 (50%) | 13 (54.2%) | 0 | 0 | - |
| No | 2 (50%) | 11 (45.8%) | 5 (100%) | 5 (100%) | - |
