# Supplementary material for: Integrated analysis of transcription factor-mRNA-miRNA regulatory network related to immune characteristics in medullary thyroid carcinoma
Source: Front Immunol. 2023 Jan 12;13:1055412. doi: 10.3389/fimmu.2022.1055412 (PMC9877459; doi:10.3389/fimmu.2022.1055412)
Supplement: Supplementary file 10 [file Table_3.doc]

| **Table S3** Primers for biomarkers in this study | | |
| --- | --- | --- |
| ID | Forward | Reverse |
| PCSK1 | CATTGTGACGGATGCTATTGAG | GTTTTCCCATCATCATTAGGGC |
| SCG2 | AATCAACGATGAGATGAAACGC | CTTCCTGCAGCATTTACTAACC |
| SNAP91 | GATGCTTTCTCCTCTCCACCACAAG | TGACACCTGAAGAATCCACCTTTGC |
| KIF5C | CTGCAACTGGAACAGGAGAAGC | GAGGTCTTCTCTGGCTTGTTCC |
| CHGB | AGAAAGCAGGCTTCAGCTATAA | CTACTCTTCTCTCGGCTATGTG |
| RAB3A | GTTCAAGATTCTCATCATCGGC | CGATAGATGGTCTTGACCTTGA |
| NEFL | TACAGACCAGCTCCTATCTGAT | AATGGTTTCCTCCACTTCGATC |
| CHGA | GCCTTGTCTCCTACTCCTGACTCC | CTGCCTGTGTTCAGAGAAGGAATCG |
| CALCA | CAGGACTATGTGCAGATGAAGG | CTCTCTTCTGGGCAATGATTCT |
| PTPRN | TCTGTATTCAGGATGGCTTGTT | GACAATCCTTGGGACATGAGTT |
| APLP1 | AAAAGGGTCCACAGAACAAGAT | CATTCACCTTTCGCTCATACTG |
| UCHL1 | AACCCCGAGATGCTGAACAAAGTG | GAGCCCAGAGACTCCTCTTCCAG |
| CDK5R2 | CAAACCTGGTGTTCGTGTAC | ATGTAGGAGTAGGCGAGGTAG |
| SV2A | TTCACCATGTCATTCAGCTACT | ATCTGATTCTCCAACGTGAAGT |
| NEFM | GGCTCTGGATATAGAAATCGCT | CCTCCACTTTGGTTTCCTCTAT |
| BATF3 | GAGGATGATGACAGGAAGGTC | TATTCCTCATGGAGCTTGTCAG |
| GMEB1 | CCCAAGTGATTTTGCAGTTACA | TAGTGCCTGTATCAATCCCTTC |
| NFIA | AGAATTTGTCCAACTTGTCTGC | CTCCTTCTGTTGACGTGGTT |
| REST | CCATCACCACCACTGCCAAAGG | TCTCATCTGCCTCTTCTGCTCCTAC |
| ZNF281 | GTTATGTTCAACCACCGTCTTC | TAGAGGAGGATAACACGCATTG |
| hsa-miR-130a-3p | CTCGACCGCAGTGCAATGTTA |  |
| hsa-miR-150-3p | ACAACAACCTGGTACAGGCCT |  |
| hsa-miR-18a-5p | AAGCGCCTTAAGGTGCATCTAG |  |
| hsa-miR-18b-5p | AAGCGCCTTAAGGTGCATCTAG |  |
| hsa-miR-193a-3p | ACATTGGCAACTGGCCTACAAA |  |
| hsa-miR-205-5p | AACAGTGTCCTTCATTCCACCG |  |
| hsa-miR-20b-5p | AATTGCTCCAAAGTGCTCATAGTG |  |
| hsa-miR-219a-5p | ACGCCGTGATTGTCCAAACG |  |
| hsa-miR-223-3p | CCTCGAGCTGTCAGTTTGTCAA |  |
| hsa-miR-31-5p | AACACGCAGGCAAGATGCTG |  |
| hsa-miR-363-3p | ACAACAACAATTGCACGGTATCC |  |
| hsa-miR-455-3p | AATTGATCGCAGTCCATGGGC |  |
| hsa-miR-584-5p | AAGTTGCATTATGGTTTGCCTGG |  |
