# Supplementary material for: Integrated analysis of transcription factor-mRNA-miRNA regulatory network related to immune characteristics in medullary thyroid carcinoma
Source: Front Immunol. 2023 Jan 12;13:1055412. doi: 10.3389/fimmu.2022.1055412 (PMC9877459; doi:10.3389/fimmu.2022.1055412)
Supplement: Supplementary file 11 [file Table_4.doc]

| **Table S4** Validation of the regulatory relationships between transcription factors and hub genes in public databases | | |
| --- | --- | --- |
| Transcription factor | Hub gene | Validation database |
| BATF3 | CALCA | Cistrome Data Browser, GRNdb |
| BATF3 | CDK5R2 | Cistrome Data Browser |
| BATF3 | CHGA | Cistrome Data Browser |
| BATF3 | CHGB | Cistrome Data Browser |
| BATF3 | KIF5C | Cistrome Data Browser, Genecards, GRNdb |
| BATF3 | NEFL | Cistrome Data Browser |
| BATF3 | PCSK1 | GRNdb |
| BATF3 | PTPRN | Cistrome Data Browser |
| BATF3 | RAB3A | Cistrome Data Browser, Genecards |
| BATF3 | SCG2 | Cistrome Data Browser |
| BATF3 | SNAP91 | Cistrome Data Browser |
| BATF3 | SV2A | Cistrome Data Browser |
| GMEB1 | CALCA | GRNdb |
| GMEB1 | CHGA | GRNdb |
| GMEB1 | CHGB | Not found |
| GMEB1 | KIF5C | GRNdb |
| GMEB1 | PCSK1 | Not found |
| GMEB1 | RAB3A | Cistrome Data Browser |
| GMEB1 | SCG2 | GRNdb, hTFtarget |
| NFIA | CHGA | Cistrome Data Browser |
| NFIA | CHGB | Cistrome Data Browser |
| NFIA | KIF5C | Cistrome Data Browser, GRNdb |
| NFIA | NEFL | Cistrome Data Browser, GRNdb |
| NFIA | NEFM | Cistrome Data Browser |
| NR4A2 | CHGB | Not found |
| NR4A2 | NEFL | Not found |
| NR4A2 | NEFM | Not found |
| NR4A2 | PCSK1 | Not found |
| NR4A2 | RAB3A | Not found |
| NR4A2 | SNAP91 | Not found |
| REST | APLP1 | Cistrome Data Browser, Genecards, hTFtarget |
| REST | CDK5R2 | Cistrome Data Browser, Genecards, GRNdb, hTFtarget |
| REST | CHGA | Cistrome Data Browser, Genecards, hTFtarget |
| REST | CHGB | Cistrome Data Browser, Genecards, GRNdb, hTFtarget |
| REST | KIF5C | Cistrome Data Browser, Genecards, GRNdb, hTFtarget |
| REST | NEFL | Cistrome Data Browser, Genecards, hTFtarget |
| REST | NEFM | Cistrome Data Browser, Genecards, GRNdb, hTFtarget |
| REST | PCSK1 | Cistrome Data Browser, Genecards, GRNdb, hTFtarget |
| REST | PTPRN | Cistrome Data Browser, Genecards, GRNdb, hTFtarget |
| REST | RAB3A | Cistrome Data Browser, Genecards, GRNdb, hTFtarget |
| REST | SCG2 | Cistrome Data Browser, Genecards, hTFtarget |
| REST | UCHL1 | Cistrome Data Browser, Genecards, GRNdb, hTFtarget |
| ZNF281 | APLP1 | Cistrome Data Browser, hTFtarget |
| ZNF281 | CDK5R2 | GRNdb |
| ZNF281 | KIF5C | Genecards |
| ZNF281 | PTPRN | Cistrome Data Browser, hTFtarget |
| ZNF281 | RAB3A | Cistrome Data Browser, GRNdb |
| ZNF281 | SV2A | Not found |
